# Assessment of Psychological Impact of Covid Pandemic on Frontline workers at Points of Entry,Pakistan: Nadia Noreen

**DOI:** 10.1093/eurpub/ckac131.472

**Published:** 2022-10-25

**Authors:** N Noreen, S Habib Ullah, S Ali Khan, A Ullah Faiz

**Affiliations:** 1 Directorate of Central Health Establishment, Ministry of National Health Regulations, Islamabad, Pakistan; 2 Health Services Academy, MOH, Islamabad, Pakistan; 3 Admin, MOH, Islamabad, Pakistan

## Abstract

**Background:**

The unprecedented public health crisis of the COVID-19 pandemic has caused heightened levels of stress and fear among health care workers.With the advent of COVID-19 in Pakistan,frontline workers of POEs have been under physical and psychological pressure including a high risk of infection, abnormal levels of workload, prolonged working hours, lack of personal protective equipment for safety from contagion, isolation, exhaustion, and lack of contact with family.The study aims to assess the impact of Covid-19 on the mental health of frontline healthcare workers.

**Methods:**

A descriptive study was conducted among HCWs across points of entry from 1st October 2020 to 31st December 2020.Data was collected using a structured questionnaire.Depression, anxiety, and stress scale (DASS-21)was used for the assessment of depression, stress, anxiety. Descriptive analysis of socio-demographic and professional factors was done. Multivariable logistic regression analysis (MLRA) was performed using SPSS version 23.0.

**Results:**

A total of 628 participants (586 males and 42 females) completed questionnaire.The mean age of the participants was 42.6 ± 45.9 years. The majority of the respondents were married (94.3%). The frequency of depression, anxiety, and stress in the HCWs was 12.1%, 42.3%, and 22.1 %, respectively. Multivariable logistic regression analysis found that the depression in HCWs was significantly associated with the profession and age (P < 0.001). The anxiety in HCWs was associated with their age and gender (P < 0.005). The stress in HCWs was significantly associated with their age (P < 0.05).

**Conclusions:**

The HCWs at the Points of entry across Pakistan showed mild to moderate symptoms of DAS. The COVID-19 pandemic has caused a heavy psychological impact among the frontline healthcare professionals. Timely psychological counseling and early psychological intervention need to be implemented for HCWs to alleviate their anxiety and stress and improve their general mental health.

**Key messages:**

• The COVID-19 pandemic has caused a heavy psychological impact.

• Timely psychological counseling and early psychological intervention.

